# 574 How Long Do We Have?: A Retrospective Review of Palliative Extubation in the Burn Unit

**DOI:** 10.1093/jbcr/iraf019.203

**Published:** 2025-04-01

**Authors:** Hannah Jones, Alexander Kurjatko, Colette Galet

**Affiliations:** University of Iowa Burn Center; University of Iowa; University of Iowa

## Abstract

**Introduction:**

Palliative extubation (PE) is the termination of mechanical ventilation as a comfort measure for imminently dying patients. Anticipating a patient’s survival time after palliative extubation is an important part of counseling patient families and can facilitate individualized palliative care and organ donation processes. While this has been studied within neurological, pediatric, surgical, and medical intensive care unit (ICU) populations, this has not been explored in burns. Prior studies have associated shorter life expectancy in patients with higher vasopressor requirements, elevated ventilator requirements, APACHE II scores, and presence of comorbid illnesses such as diabetes. This study was performed to identify factors associated with death within one hour of palliative extubation within our adult burn unit population.

**Methods:**

This is a retrospective cohort study. Adult patients admitted to our burn unit who underwent PE from 7/10/2015 to 6/30/2023 were identified in our institution’s burn registry. Patients who died prior to extubation were excluded. Demographics, comorbidities, injuries, and clinical parameters were collected. Variables with a p-value ≤ 0.1 in univariate analysis as well as age, sex, and TBSA (%) were included in multivariate models. Binary logistic regression was conducted using the forward Wald approach. P< 0.05 was considered significant.

**Results:**

A total of 47 patients underwent PE within the study period. Of these, 25 (53.2%) died within 60 minutes of PE. There were no significant differences in age, gender, total body surface area burned, or presence of inhalation injury. In univariate analysis, higher vasopressor requirements, higher SOFA score, larger anion gap, higher phosphorus, higher lactic acid level, lower mean arterial pressure, acidosis, and the absence of a history of cerebrovascular disease were associated with death less than 60 minutes after PE with p ≤ 0.01. Bivariate logistic regression demonstrated that higher SOFA scores (OR = 2.851 [95% CI 1.173-6.931]) and anion gaps (OR = 1.687 [95% CI 1.014-2.806]) were associated with death within 60 minutes of PE.

**Conclusions:**

Higher SOFA score and anion gap were associated with death within 60 minutes after PE. This aligns with prior literature correlating time to death with illness severity scales. While some uncertainty will always be present when predicting time to death after PE, our study provides a guide to be used in family discussions.

**Applicability of Research to Practice:**

Patients with higher SOFA scores and anion gaps may be more likely to die earlier following PE. Further studies are warranted to evaluate the predictive value of these variables.

**Funding for the Study:**

N/A

